# Supplementary figures and images for: Improved gene regulatory network inference from single cell data with dropout augmentation
Source: PLoS Comput Biol. 2025 Oct 24;21(10):e1013603. doi: 10.1371/journal.pcbi.1013603 (PMC12574904; doi:10.1371/journal.pcbi.1013603)

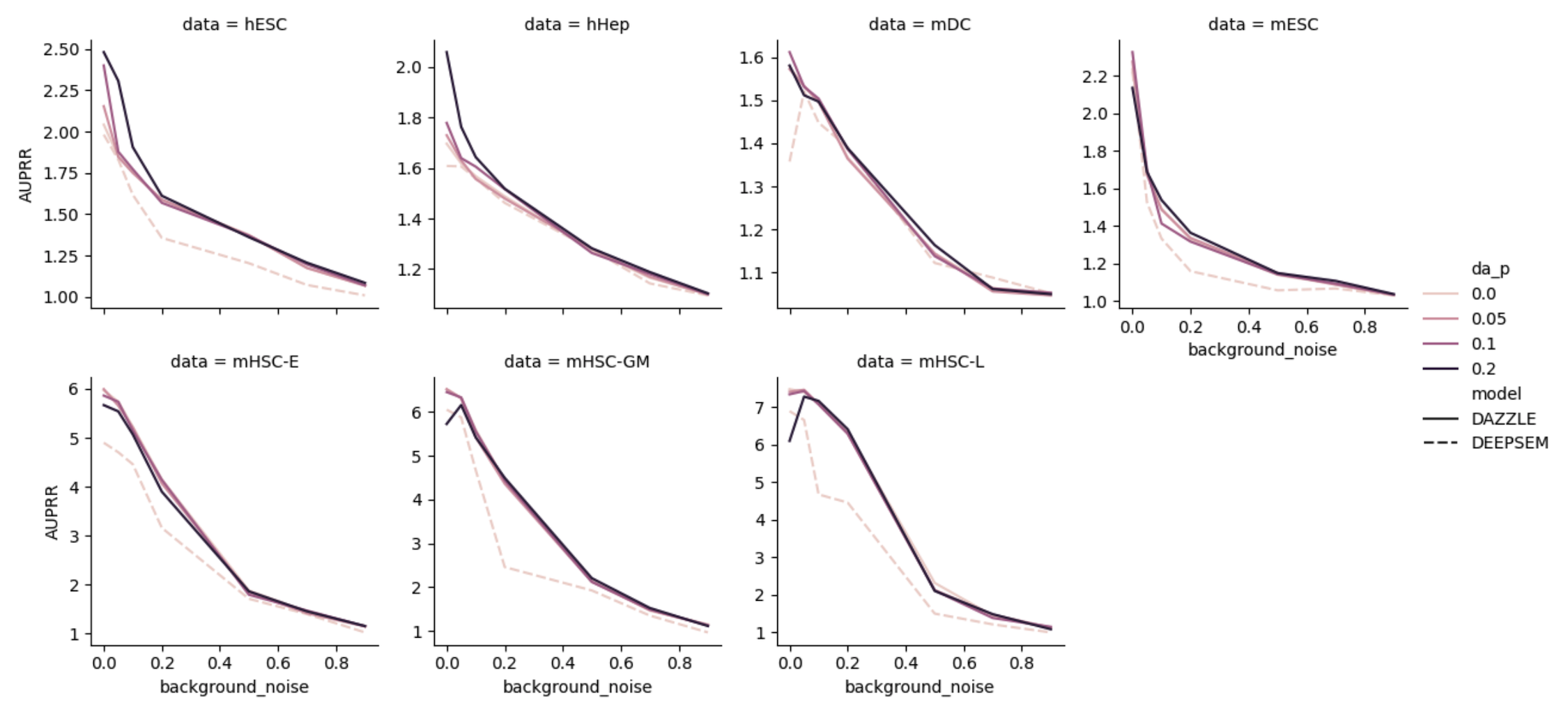

Supplement: S1 Fig — Certain proportions of data points (x-axis) were drop to simulate background dropout noise at the very beginning. (TIFF) [file pcbi.1013603.s001.tiff]

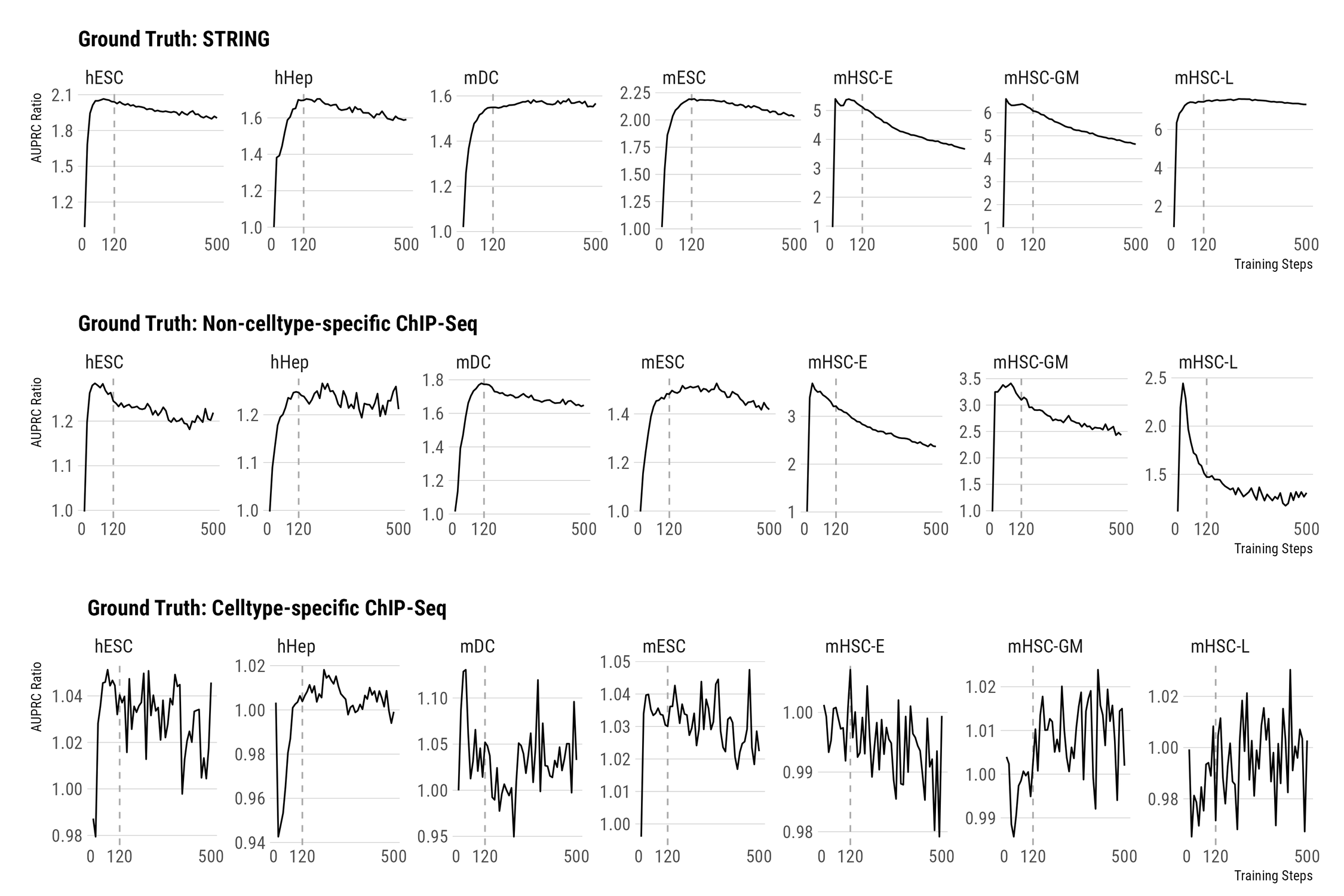

Supplement: S2 Fig — Quality may quickly downgrade after the performance peak. Dashed line is the recommended stopping point from DeepSEM. Note that for the celltype-specific data sets, performance is particularly volatile. (TIFF) [file pcbi.1013603.s002.tif]

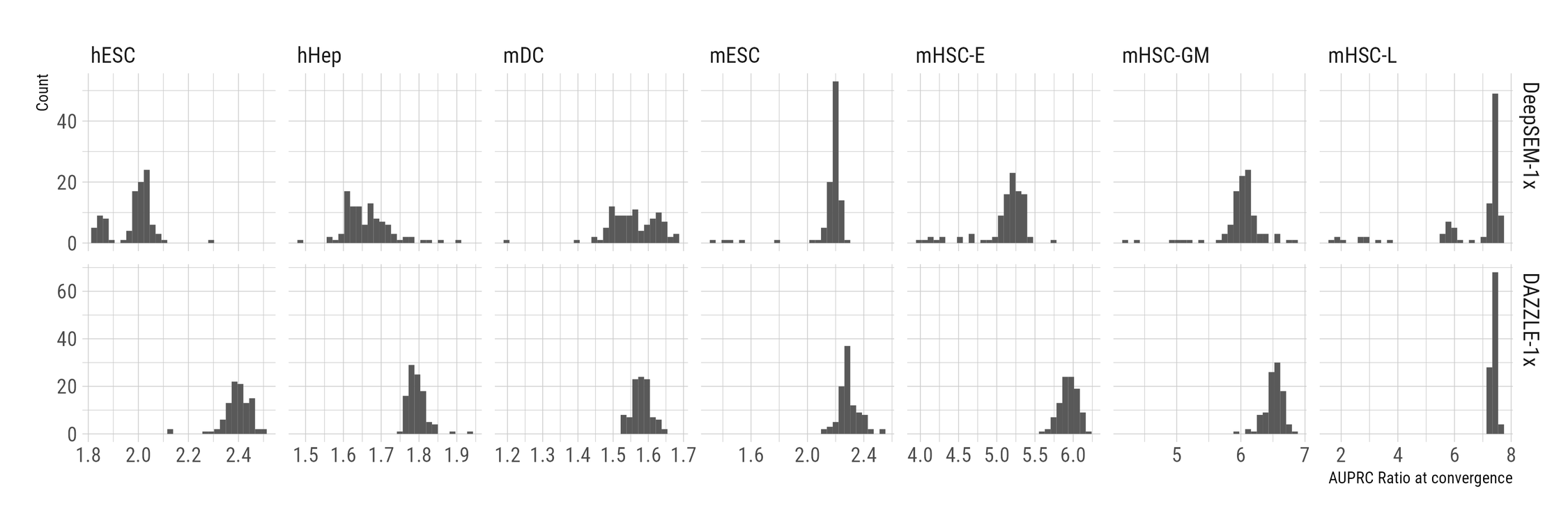

Supplement: S3 Fig — Results from DAZZLE-1x tend to be more stable than results from DeepSEM-1x. (TIFF) [file pcbi.1013603.s003.tif]
