# Supplementary material for: Improved gene regulatory network inference from single cell data with dropout augmentation
Source: PLoS Comput Biol. 2025 Oct 24;21(10):e1013603. doi: 10.1371/journal.pcbi.1013603 (PMC12574904; doi:10.1371/journal.pcbi.1013603)
Supplement: S1 Text — (PDF) [file pcbi.1013603.s004.pdf]

Supplements for Dropout Augmentation and DAZZLE

1 Ablation study

To further study the actual impact of DA and DAZZLE, we conducted a controlled comparison on the BEELINE benchmarks (with STRING as ground truth) by training variants of DAZZLE-1x (with different DA probabilities) and DeepSEM-1x under identical hyperparameter settings (NN learning rate = 1×10^{-4}, Adj matrix learning rate = 2×10^{-5}, batch size = 64). In all cases, we zeroed out a certain proportion of data points before the models saw the data to simulate background dropout noise; trials with background noise rates of zero correspond to the original data. Note that this "background noise" is different from DA as used in DAZZLE since in DA, the dropouts are sampled independently in every iteration and the model eventually sees them all. As shown in Figure S1, DAZZLE-1x (solid lines) maintains its performance advantages compared to DeepSEM-1x (dashed line) in nearly all cases. In many cases, such as hESC, hHep, mDC, mHSC-E, mHSC-GM, and mHSC-L, DAZZLE-1x with 10% additional background dropout noise still performs better than DeepSEM-1x with the full data. Note that when the background dropout noise is very high ($> 50\%$), different DA probabilities seem to yield similar performance. We believe a fair explanation here is that when the amount of useful information is too limited, the model is not able to learn any insightful knowledge. In that case, making the model more robust does not offer any advantages.

2 Comparison of Hyperparameter Searching between DAZZLE and DeepSEM

There are concerns that the performance gain on DAZZLE came from better hyperparameter searching. However, the fact is that in DAZZLE, to make the comparison easier, we are using the exact same choices of hyperparameters as used in DeepSEM except for the ones we described in the main paper (Dropout augmentation probability, delayed steps on sparse, number of optimizers). We could probably get slightly better performances by using another set of hyperparameters but that's not the purpose of this paper. Also, DeepSEM has already chosen a set of hyperparameters that offer good performances.

Table A. Comparison of Hyperparameter Choices between DAZZLE and DeepSEM

	DAZZLE	DeepSEM
train_split	1	1
train_split_seed	None	None
hidden_dim	128	128
z_dim	1	1
train_on_non_zero	FALSE	FALSE
dropout_augmentation_p	0.1	0
alpha	100	100
beta	1	1
chi	0.5	0
h_scale	0	0
delayed_steps_on_sparse	5	0
number_of_opt	1	2
batch_size	64	64
n_epochs	120	120
eval_on_n_steps	10	10
early_stopping	0	0
lr_nn	0.0001	0.0001
lr_adj	0.00002	0.00002
K1	1	1
K2	2	2

3 Performance on additional benchmarks

Table B. Performance on Additional Benchmarks

	hESC	hHep	mDC	mESC	mHSC-E	mHSC-GM	mHSC-L
# of Genes	1410	1448	1321	1620	1204	1132	692
# of Cells	758	425	383	421	1071	889	847
**Metric: Early Precision Ratio; Ground Truth: STRING**							
# of True Edges	5,149	9,000	5,898	8,479	1,826	1,311	154
GENIE3	3.66 (0.07)	3.17 (0.03)	2.19 (0.04)	3.48 (0.03)	7.00 (0.06)	8.53 (0.09)	7.34 (0.14)
GRNBoost2	3.46 (0.09)	2.78 (0.04)	2.08 (0.04)	3.69 (0.05)	6.21 (0.06)	7.32 (0.12)	7.12 (0.24)
PIDC	4.13 (0.00)	*3.63 (0.00)*	1.87 (0.00)	3.68 (0.00)	*8.10 (0.00)*	8.39 (0.00)	6.53 (0.00)
DeepSEM-1x	4.23 (0.12)	3.12 (0.18)	2.06 (0.09)	3.89 (0.10)	7.36 (0.22)	7.91 (0.33)	7.21 (0.47)
DeepSEM-10x	4.54 (0.11)	3.58 (0.18)	*2.20 (0.07)*	*4.15 (0.07)*	7.86 (0.07)	*8.96 (0.19)*	*7.43 (0.20)*
DAZZLE-1x	5.12 (0.14)	3.25 (0.08)	2.02 (0.05)	3.90 (0.08)	7.79 (0.17)	8.54 (0.15)	7.37 (0.13)
DAZZLE-10x	*5.25 (0.05)*	3.33 (0.03)	2.01 (0.03)	3.96 (0.07)	8.06 (0.06)	8.53 (0.08)	7.40 (0.07)
**Metric: Early Precision Ratio; Ground Truth: Non-celltype-specific ChIP-Seq**							
# of True Edges	4,597	5,335	3,918	8,030	1,960	1,358	317
GENIE3	1.68 (0.03)	1.85 (0.08)	2.93 (0.08)	3.47 (0.05)	4.64 (0.07)	5.86 (0.09)	3.05 (0.09)
GRNBoost2	1.49 (0.05)	1.70 (0.05)	2.58 (0.07)	*3.51 (0.05)*	4.71 (0.11)	5.03 (0.15)	3.02 (0.11)
PIDC	2.08 (0.00)	2.44 (0.00)	2.95 (0.00)	2.80 (0.00)	5.75 (0.00)	5.86 (0.00)	2.99 (0.00)
DeepSEM-1x	2.20 (0.19)	2.56 (0.12)	3.29 (0.14)	3.31 (0.12)	5.55 (0.39)	5.66 (0.32)	3.14 (0.28)
DeepSEM-10x	2.18 (0.05)	2.67 (0.07)	*3.73 (0.08)*	3.50 (0.06)	6.04 (0.14)	6.13 (0.22)	*3.35 (0.16)*
DAZZLE-1x	2.31 (0.12)	2.77 (0.16)	3.51 (0.11)	3.21 (0.14)	6.29 (0.13)	6.11 (0.11)	3.30 (0.17)
DAZZLE-10x	*2.40 (0.04)*	*2.83 (0.05)*	3.64 (0.07)	3.26 (0.04)	*6.36 (0.06)*	*6.21 (0.05)*	3.34 (0.06)
**Metric: Early Precision Ratio; Ground Truth: Celltype-specific ChIP-Seq**							
# of True Edges	7,050	15,410	1,193	42,795	21,975	14,135	5,180
GENIE3	0.95 (0.01)	*1.12 (0.01)*	0.99 (0.06)	*1.06 (0.00)*	*1.02 (0.00)*	1.03 (0.09)	*1.07 (0.00)*
GRNBoost2	0.78 (0.02)	0.27 (0.00)	0.97 (0.08)	0.33 (0.00)	0.21 (0.00)	0.23 (0.01)	0.44 (0.01)
PIDC	0.92 (0.00)	1.01 (0.00)	1.10 (0.00)	1.01 (0.00)	0.97 (0.00)	0.98 (0.00)	0.99 (0.00)
DeepSEM-1x	1.13 (0.04)	1.03 (0.01)	1.11 (0.07)	1.02 (0.01)	1.01 (0.00)	1.02 (0.00)	1.04 (0.01)
DeepSEM-10x	1.15 (0.02)	1.04 (0.00)	1.15 (0.03)	1.02 (0.01)	1.01 (0.00)	*1.03 (0.00)*	1.04 (0.00)
DAZZLE-1x	1.20 (0.03)	1.03 (0.01)	1.11 (0.06)	1.02 (0.01)	1.01 (0.00)	1.02 (0.00)	1.06 (0.01)
DAZZLE-10x	*1.20 (0.01)*	1.03 (0.00)	*1.17 (0.02)*	1.02 (0.00)	1.01 (0.00)	1.03 (0.00)	1.06 (0.00)

Number of target genes: 1000.
Higher ratios indicate better performance. Here, italicized cell in dark shades indicate the best algorithms and lightly shaded cell indicate the 2nd best algorithm.

Table C. Performance Comparison of DAZZLE-1x and DeepSEM by AUROC.

	hESC	hHep	mDC	mESC	mHSC-E	mHSC-GM	mHSC-L
# of Genes	1410	1448	1321	1620	1204	1132	692
# of Cells	758	425	383	421	1071	889	847
**STRING**							
# of True Edges	5,149	9,000	5,898	8,479	1,826	1,311	154
DeepSEM-1x	0.616 (0.003)	0.590 (0.006)	0.555 (0.003)	0.575 (0.002)	0.646 (0.003)	0.683 (0.019)	0.677 (0.063)
DAZZLE-1x	0.638 (0.002)	0.609 (0.001)	0.569 (0.004)	0.607 (0.002)	0.663 (0.005)	0.704 (0.000)	0.703 (0.006)
**Non-celltype-specific ChIP-Seq**							
# of True Edges	4,597	5,335	3,918	8,030	1,960	1,358	317
DeepSEM-1x	0.524 (0.010)	0.532 (0.017)	0.556 (0.001)	0.563 (0.004)	0.596 (0.007)	0.611 (0.024)	0.584 (0.043)
DAZZLE-1x	0.543 (0.007)	0.547 (0.003)	0.566 (0.004)	0.570 (0.002)	0.588 (0.003)	0.603 (0.005)	0.618 (0.001)
**Celltype-specific ChIP-Seq**							
# of True Edges	7,050	15,410	1,193	42,795	21,975	14,135	5,180
DeepSEM-1x	0.508 (0.012)	0.510 (0.014)	0.501 (0.011)	0.515 (0.004)	0.489 (0.014)	0.489 (0.022)	0.512 (0.026)
DAZZLE-1x	0.509 (0.007)	0.503 (0.011)	0.506 (0.006)	0.512 (0.003)	0.494 (0.007)	0.517 (0.004)	0.548 (0.006)

Tests on this set of evaluation are repeated 3 times. Results show similar trend as results evaluated in AUPRC and EP.
