# Supplementary material for: Improved gene regulatory network inference from single cell data with dropout augmentation
Source: PLoS Comput Biol. 2025 Oct 24;21(10):e1013603. doi: 10.1371/journal.pcbi.1013603 (PMC12574904; doi:10.1371/journal.pcbi.1013603)
Supplement: Attachment — Submitted filename: DAZZLE_response_to_reviewers_5.31.pdf [file pcbi.1013603.s005.pdf]

Tufts UNIVERSITY | DEPARTMENT OF COMPUTER SCIENCE

Response to Reviewer #1:

1. **DAZZLE performs better than DeepSEM. Why can you claim that the improvement is from solving the zero inflation problem?**

   This is a valid question. Our claim was previously supported by the following evidence: In Figure 2, we demonstrate that introducing a controlled amount of dropout augmentation (DA) while holding everything else constant leads to a slower decline in performance when the training continues. This indicates that DA does help prevent model overfitting to the irrelevant noise patterns in the data.

   With this revision, we made the following edits to the paper:

   - We followed your suggestions and added the suggested experiment, as shown in the supplement section 1.
   - We also edited the first paragraph of the Discussion (Section 4) and reviewed the theoretical background of DA. We further discussed the expectation of running DA for other use cases in single cell analyses.

2. **How does the method work when applied to data that are not stationary?**

   We appreciate this insightful suggestion. We acknowledge the reviewer's concern about applying both DAZZLE and DeepSEM to non-stationary data since both methods are based on the SEM assumption. However, there is often a difference between developmental data and non-stationary data. In our case study on mouse microglia, we only have 5 developmental stages (embryonic, and 5/30/100/540 days after birth), and each developmental stage was analyzed independently as a cross-sectional snapshot. We then validated the trends of top regulators with existing literature. We believe this approach is justifiable because the number of time points is very limited. At least 2 time points are from adult mice, and in those cases the GRNs are in fact quite stationary. In practice, we find that the embryonic stage is the only stage where this issue may debatably arise. However, it is also common practice to analyze GRNs in embryonic cells with "stationary methods." For example, the hESC and mESC cells covered in the BEELINE benchmark are both embryonic stem cells. So this comment applies to many other publications as well.

   In this revision, we edited the first paragraph of Section 3.2.2 on the Hammond microglia data and made it clear that we run the analysis as cross-sectional slices.

3. **Hyperparameter tuning and concerns about overfitting**

Please see Supplement section 2 for a table comparing hyperparameters in DAZZLE and DEEPSEM. In fact, in DAZZLE, to make the comparison easier, we are using the exact same choices of hyperparameters as used in DEEPSEM except for the ones we described in the main paper (Dropout augmentation probability, delayed steps on sparse, number of optimizers). We could probably get slightly better performance by using another set of hyperparameters but that's not the purpose of this paper. Also, DEEPSEM has already chosen a set of hyperparameters that offer good performance.

4. **Novelty and impact**

Single cell data is known for its abundance of dropout noise, yet the most prevent methods to solve this problem are through data imputation (scImpute, DeepImpute, etc) and autoencoders. While these methods do work in their ways, they often require additional assumptions and may introduce unwanted biases. Dropout augmentation follows the theory of classic noise injection and provides a fresh solution to address the dropout problem in single-cell data without any additional assumptions and data. Since this simple method is widely used in machine learning, it's reasonable to believe that the idea of dropout augmentation may also generate impact in other applications of single cell analyses.

5. **Clarification of input data types and inference capabilities**

Thank you for making these points! The input of the model is simply a log-transformed gene expression matrix, where rows are cells and columns are genes. We have added this explanation to section 2.1.

6. **Use PIDC as a baseline method**

We agree that PIDC is a strong baseline method, and we would love to include that for comparison. However, as of today, this package is no longer available.

7. **Why not evaluate using AUROC?**

The number of edges is usually significantly smaller than the number of non-edges. For example, in the hESC dataset used in this study, while there are 5,149 edges, there are 1,410 x 410 = 578,100 potential edges. As mentioned in Section 3.4, we

have a significant class imbalance. It makes more sense to use AUPR instead of AUROC, which is why why BEELINE recommended AUPR Ratio and EPR in their paper. However, for easy comparison with other methods, in this revision we provide a small comparison between DeepSEM-1x and DAZZLE-1x in Supplement Table S3. The trend is similar to the results reported in AUPRR and EPR.

8. **Distinction between positive and negative regulation**

One of the advantages of DEEPSEM and DAZZLE is that they use neural networks to do nonlinear transformation. At the end, the learned adjacency matrix is in fact describing the relationships among the non-linearly transformed data. Therefore, positive/negative regulation is not directly computable. One way to work around is that after the existence of regulation is confirmed, we can create another simple linear model between the TF and the target and use the sign of the linear model as the sign of the inferred relationship. We have added this explanation to the end of the Discussion section

Response to Reviewer #2

1. **Extension to multi-omics data**

Thanks for this suggestion! Yes, we agree with the reviewer that using multi-omics data can significantly boost the performance of GRN inference. In fact, there are several popular frameworks, such as SCENIC+, that integrate data types to build atop transcriptomics-based GRNs. Results from DAZZLE can be easily integrated into these frameworks as e.g. a replacement for GRNBoost2. We add some discussion to the Discussion section.

2. **Please clarify the role of DA classifier**

The DA classifier is designed to tag augmented zeros in the latent space so that the decoder can learn to discount similar looking data and reduce the negative impact of dropout.

3. **Further analysis on how DA improves GRN inference**

In the previous version of the manuscript, we have demonstrated empirical evidence to explain how DA alone improves the outcome of GRN inference in Figure 2. We also have discussed the connection with existing theoretical justifications in machine learning/deep learning research.

In this revision,

- We followed the suggestions from you and added the suggested experiment as shown in the supplement section 1.
- We also edited the first paragraph of Discussion (Section 4) and reviewed the theoretical background of DA. We also discussed the expectation of running DA for other use cases in single cell analyses.

4. **Theoretical and empirical justifications for [the results in Table 1] are needed.**

   In sections 2.3 and 2.4 (Figures 2 and 3), we have discussed and provided empirical experiments to explain the improvement in DAZZLE's performance from two different perspectives. We have also discussed theoretical justification in the main text. In this revision, we have edited the caption of Table 1 to make it more clear.

5. **Compare to newer methods as well.**
   The main point of this paper is dropout augmentation instead of the DAZZLE method. We have proposed a followup work named RegDiffusion with better performance. This has already been published, and refers to the DAZZLE preprint.

6. **AUROC, F1**

   The number of edges is usually significantly smaller than the number of non-edges. For example, in the hESC dataset used in this study, while there are 5,149 edges, there are 1,410 x 410 = 578,100 potentially edges. As mentioned in Section 3.4, we have a significant class imbalance. It makes more sense to use AUPR instead of AUROC which is why BEELINE recommended AUPR Ratio and EPR in their paper. However, for easy comparison with other methods, in this revision we provide a small comparison between DeepSEM-1x and DAZZLE-1x in Supplement Table S3. The trend is similar to the results reported in AUPRR and EPR.

Response to Reviewer #3

1. **Combine DA with methods other than DeepSEM**

   This is a nice suggestion, since our primary focus here was to demonstrate the effectiveness of DA. DeepSEM is an established GRN inference framework despite its well-known problem with stability. (We do take some issue with calling a 2021 method "relatively old" for a manuscript we submitted in 2024, when DeepSEM was still a typically-used comparator, see e.g. *Cell Res.* 2024 Oct 8;34(12):830–845.) After improving DeepSEM's stability, the method offers a clear baseline for

comparison, so we can observe how DA reduces overfitting to dropout noise. We appreciate that combining DA with other methods could be one of the future directions for this work.

2. AUROC

The number of edges is usually significantly smaller than the number of non-edges. For example, in the hESC dataset used in this study, while there are 5,149 edges, there are 1,410 x 410 = 578,100 potentially edges. As mentioned in Section 3.4, we are having a significant class imbalance. It makes more sense to use AUPR instead of AUROC and that's why BEELINE recommended AUPR Ratio and EPR in their paper. However, for easy comparison with other methods, in this revision we provide a small comparison between DeepSEM-1x and DAZZLE-1x in Supplement Table S3. The trend is similar to the results reported in AUPRR and EPR.

3. More recent baseline methods

The main point of this paper is dropout augmentation instead of the DAZZLE method. We have proposed a follow-up work named RegDiffusion with better performance.

322 Joyce Cummings Center, 177 College Avenue, Medford, MA 02155
