# Supplementary material for: Improved gene regulatory network inference from single cell data with dropout augmentation
Source: PLoS Comput Biol. 2025 Oct 24;21(10):e1013603. doi: 10.1371/journal.pcbi.1013603 (PMC12574904; doi:10.1371/journal.pcbi.1013603)
Supplement: Attachment — Submitted filename: response_to_reviewers_dazzle.pdf [file pcbi.1013603.s006.pdf]

Dear Editor and Reviewers,

Thank you for your time and for the insightful comments on our manuscript, “Improved gene regulatory network inference from single cell data with dropout augmentation”. We appreciate the constructive feedback, which has helped us significantly improve the quality and clarity of our paper. We have carefully addressed all the points raised and have revised the manuscript accordingly.

Below, we provide a point-by-point response to the comments.

Response to Reviewer 1

1. *“DA and zero inflation: Figure S1 shows that with or without zero inflation, DA can improve DeepSEM. However, DA cannot fully cancel the effect of zero inflation. The authors should lower the tone. Instead of the false impression that “DA can solve the zero inflation problem”, it is better to state that DA can improve performance even with zero inflation.”*

   We agree with the reviewer that our original phrasing was too strong. We have toned down our claims in the Abstract and Introduction to more accurately state that data augmentation (DA) improves the model's performance in the presence of zero-inflation, rather than completely solving the problem. Furthermore, to more directly address this point, we have included the reviewer's proposed ablation study. This analysis (new in the prior revision) demonstrated that DAZZLE is indeed more robust and handles dropout events more effectively than the prior model, providing stronger evidence for our revised claims. We appreciate this suggestion.

2. *“Explain that each entry of the gene expression matrix represents the scRNAseq result, where the original value x is replaced by log(1+x). Otherwise, readers might wonder how 0 is handled.”*

   We thank the reviewer for this excellent suggestion for improving clarity. We have added text in Section 2.1 to explicitly state that the gene expression matrix is transformed using log(1+x) to handle zero values, as in common practice.

3. *“PIDC method has a GitHub page:https://github.com/Tchanders/network_inference_tutorials. What do you mean by “this package is no longer available”?””*

   We apologize for the previous incorrect comment. We meant to say that the PIDC R package was no longer available (https://rdrr.io/github/hmutpw/PIDC/; apparently it was not an official implementation either). We sincerely thank the reviewer for providing the correct link for the Julia software. In this revision, we have incorporated PIDC as one of the baseline methods in our comparisons. The

method is described in the Introduction, and PIDC has been added to all relevant figures and tables throughout the Results section and the Supplement. While it is indeed a strong method and performs competitively in some cases, it does not change any of the conclusions about DA in the manuscript.

Response to Reviewer 2

We thank Reviewer 2 for their positive feedback on our work.

Response to Reviewer 4

1. *"The authors claimed improved efficiency and reduced computational time for DAZZLE... While it may not be feasible to provide detailed computational time or GPU specs, the authors could strengthen their claim by including example run times or comparative results to illustrate the efficiency gains."*

   We thank the reviewer for this excellent suggestion for improving clarity. To substantiate our claims of improved efficiency, we have added a concrete example of comparative run times for DAZZLE and DeepSEM in Section 2.1. This example illustrates the practical computational savings offered by our method.

2. *"For datasets with a large number of genes and cells, a single pass of DeepSEM or DAZZLE can take several hours to compute... In such cases, a single pass of DAZZLE offers a sufficiently-good solution at a more reasonable cost." It is unclear whether the first mention of DAZZLE refers to DAZZLE-10x and the second to DAZZLE-1x. If so, please clarify...*

   We thank the reviewer for pointing out this ambiguity. The original text was indeed confusing. We have revised this paragraph to clearly state that we are discussing DAZZLE-1x in this case.

3. **"***In Section 2.5, in addition to literature evidence, the authors may consider databases such as TRRUST v2 and TFLink to query experimentally validated TF-gene interactions. This would allow for a more quantitative evaluation of DAZZLE's performance by reporting how many ground truth TF-gene interactions were identified."*

   We thank the reviewer for this excellent suggestion and for the opportunity to clarify our evaluation strategy. We completely agree on the importance of quantitative evaluation using databases like TRRUST v2. In fact, we already use TRRUST v2, along with DoRothEA and RegNetwork (from BEELINE), data: they were used to construct the comprehensive, non-cell-type-specific ChIP-Seq benchmark used for our main quantitative evaluation, as detailed in Section 3.3.1 and summarized in the 2nd section of Table 1. The reason we focused on literature-based evidence specifically for the case study in Section 2.5 is due to

context specificity. While links in databases like TRRUST v2 and TFLink are experimentally validated, they represent a general, tissue/environment/developmental-independent “ground-truth” and do not necessarily indicate that an interaction is active in the specific context. Therefore, we believe that for the case study, curated literature evidence provides a more relevant validation of the specific case we were analyzing. To ensure clarity, we have added a brief explanation to Section 2.5 clarifying why literature evidence was chosen.

4. **“***DeepSEM and DEEPSEM were used interchangeably in the manuscript.”*

   We apologize for the inconsistency. We have corrected this throughout the manuscript to use "DeepSEM" consistently.

5. *Line 137, “celltype-specific ChiP-Seq” should be “celltype-specific ChIP-Seq”.*

   This typo has been corrected.

6. *Lines 454-460, references are needed for this paragraph. For example, cisTarget and AUCell scores haven’t been mentioned in the manuscript.*

   We thank the reviewer for noting this omission. We have added the appropriate references for cisTarget, AUCell, and the related concepts in this paragraph.

We believe the manuscript is now substantially improved and hope it is now suitable for publication.

Sincerely,

Hao Zhu, Donna Slonim
